# Synovial myxoma with cyst formation in the hip joint of a central bearded dragon

**DOI:** 10.1177/10406387241277230

**Published:** 2024-09-12

**Authors:** Melina Rasper-Hössinger, Patricia Edith Kunze, David Schmid, Eva Dervas

**Affiliations:** Institute of Veterinary Pathology, Vetsuisse Faculty, University of Zurich, Zurich, Switzerland; Clinic for Zoo Animals, Exotic Pets and Wildlife, Vetsuisse Faculty, University of Zurich, Zurich, Switzerland; Clinic of Diagnostic Imaging, Vetsuisse Faculty, University of Zurich, Zurich, Switzerland; Institute of Veterinary Pathology, Vetsuisse Faculty, University of Zurich, Zurich, Switzerland

**Keywords:** arthropathy, bearded dragon, degenerative joint disease, metabolic bone disease, neoplasia, reptile, synovial myxoma

## Abstract

Degenerative bone lesions are rarely described in reptiles and belong mainly to the broad spectrum of metabolic bone diseases. Here we describe a 7-y-old female central bearded dragon (*Pogona vitticeps*) with a complex unilateral neoplastic lesion in the hip joint. The animal was presented because of severe progressive swelling of the left hindlimb, apathy, and weight loss. The swelling was soft and surrounded the left femur. Full-body radiographs were performed in 2 orthogonal projections. The main radiologic findings were severe soft tissue swelling centered on the proximal third of the left femur and an absent left femoral head. The caretaker elected euthanasia, and a postmortem examination was performed, followed by subsequent histologic examination. The swelling consisted of variably sized myxomatous proliferations and cysts that invaded the femoral bone. Furthermore, several long bones had lesions consistent with metabolic bone and degenerative joint diseases. Synovial myxomas are rare lesions of the joints that have, to our knowledge, not been described previously in reptiles.

A ~7-y-old, intact female, captive-born central bearded dragon (*Pogona vitticeps*) was presented to the Clinic for Zoo Animals, Exotic Pets and Wildlife at the Vetsuisse Faculty, University of Zurich (Zurich, Switzerland) because of progressive apathy and weight loss. The animal had a slowly progressive swelling of the left hindleg over the last 2 y, which was initially treated with antibiotics by another veterinarian without improvement. Other pre-existing conditions included a mandibular symphysis fracture 3 y earlier that was conservatively treated with fasting for 3 wk. This animal had been acquired 3 y earlier from a private caretaker with suboptimal husbandry conditions and was currently housed in an indoor terrarium with 4 other individuals, all females. Although the exact origin of the animals was not known, 2 of the other animals had a clinical history with suspicion of metabolic bone disease (MBD), as indicated by the chronic mandibular fracture and circumferential soft tissue swellings along the limbs (aged 7 and 8 y, respectively). General husbandry, including ultraviolet B exposure, photoperiod, humidity, and temperature gradients, was considered appropriate for the species. Diet consisted of daily leafy greens (including dandelion [*Taraxacum officinale*], vetch [*Vicia sativa*], and varied herbs) and twice-weekly insects (including house crickets [*Acheta domesticus*], yellow mealworms [*Tenebrio molitor*], and desert locust [*Schistocerca gregaria*]), dusted with an unknown mineral and vitamin supplement.

Clinical examination revealed a poor body condition with a body weight of 302 g. An instability was present at the mandibular symphysis with moderate firm swelling of the mandible. Severe soft-tissue swelling was present on the left thigh; however, a distinct mass could not be palpated. The swelling was soft, without crepitus, and involved the whole femur. Pain and motor functions were present in the limb, and lameness was obvious, with the individual predominantly dragging the limb during ambulation.

Full-body radiographs were performed in latero-lateral and dorsoventral projections. The main finding was focal, severe soft-tissue swelling centered on the proximal third of the left femur and inguinal region, with several small, well-defined, speckles of mineral opacity superimposed on the swelling. The left femoral head was absent, and the femur and acetabulum had a mildly irregular surface without signs of an aggressive bone lesion (Fig. 1A). Additionally, the diameters of the right tibia-fibula and humerus were increased in the proximal third of the diaphysis, with mildly irregular thickness of the cortex and mild axis deformation, which were considered highly suspicious for chronic pathologic fractures. In the area of the rostral third of the mandible, the bone had focal, moderate moth-eaten bone lysis and mild irregular periosteal reaction suspicious of osteomyelitis.

**Figure 1.**
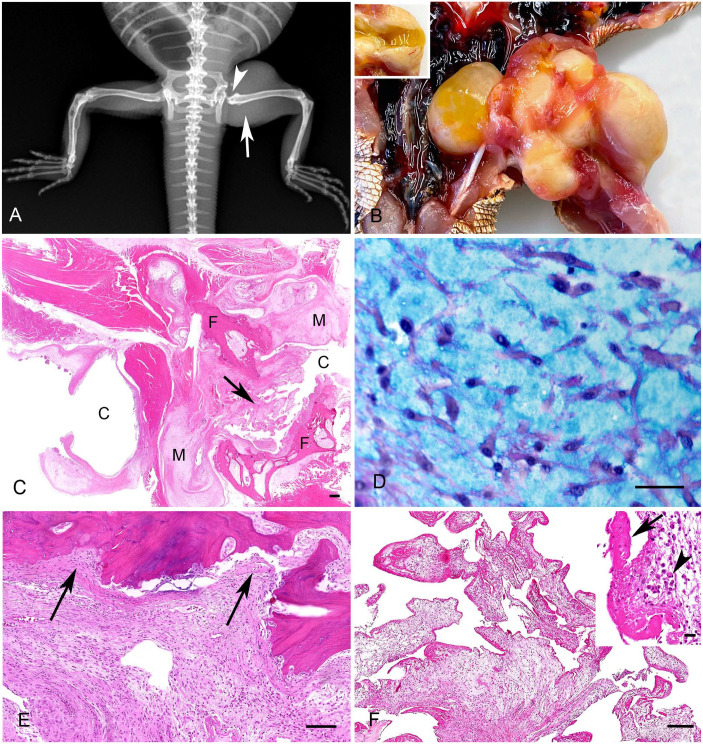
Myxoma in the left femoral head of a central bearded dragon (*Pogona vitticeps*). **A.** The main radiographic findings were severe soft-tissue swelling centered on the left femur (arrow) and the absent left femoral head (arrowhead). **B.** Grossly, the left femoral head was replaced by a moderately well-demarcated yellow mass, consisting of fluid-filled cysts and nodules. Inset: the cysts contained moderate amounts of transparent, viscous fluid resembling synovial fluid. **C.** Histologically, the mass consists of variably sized cysts (C) and nodular myxoid (M) components. Note the villus proliferations of the synovium protruding into a cyst lumen (arrow). The architecture of the bone and hip joint is entirely lost, and fragments of the femoral bone (F) are seen. H&E. Bar = 100 µm. **D.** The solid areas consist of well-differentiated stellate cells in a myxomatous matrix. PAS–alcian blue. Bar = 30 µm. **E.** The myxoid proliferations (arrows) invade trabecular bone. H&E. Bar = 100 µm. **F.** The cystic spaces are lined by a layer of cells resembling synovium that occasionally form villus-like proliferations. Bar = 250 µm. Inset: the villi are often covered by a layer of fibrin (arrow); abundant foamy macrophages (arrowhead) are seen in the matrix. Bar = 20 µm. H&E.

Given the poor prognosis, the caretaker elected euthanasia. Upon postmortem examination, the left femoral head and acetabulum were found to be completely replaced by a moderately demarcated, irregular 3 × 2 × 1-cm, soft yellow mass consisting of several variably sized cysts and solid nodules (Fig. 1B). Cysts contained a moderate amount of yellow, viscous-to-mucinous, transparent material, resembling synovial fluid, and had a thin, yellow-beige wall (Fig. 1B). Further gross lesions included moderate yellow, transparent celomic effusion, serous atrophy of the intracelomic fat and bone marrow (cachexia), and a multinodular 1 × 1 × 0.5-cm firm white mass protruding from the visceral surface of the liver. We additionally took a swab of the femoral head mass for bacterial culture to exclude bacterial causes.

We collected tissue samples from all major organs and the long bones. Samples were fixed in 10% neutral-buffered formalin, trimmed, and processed routinely. Consecutive sections (4–5 μm) were prepared and stained with H&E. A combined periodic acid–Schiff (PAS) and alcian blue stain was performed on the bone lesions to detect fungal structures and to stain the myxomatous matrix. Before histologic processing, long bones were demineralized (RDF; CellPath).

Histologically, the femoral mass had both solid and cystic areas, the former consisting predominantly of myxoid material (Fig. 1C). These solid areas formed distinct nodules or bordered and surrounded the cystic spaces. They consisted of a few single, spindle-to-polygonal (stellate) cells embedded in abundant pale basophilic-to-amphophilic, loose, myxoid material. The cells had moderate amounts of eosinophilic cytoplasm, single basophilic, central-to-peripheral, oval-to-spindle nuclei with fine chromatin, and up to 2 indistinct nucleoli (Fig. 1D). Small vessels and infiltrates of a few lymphocytes, foamy macrophages, and heterophilic granulocytes were also seen. Anisocytosis, anisokaryosis, and mitotic count were low (<1 in in 2.37 mm^2^, 10 hpfs, 40× objective, 10× ocular field number [FN] 22 mm). Between the myxoid proliferations were variably sized islands of cartilage or mineralized and non-mineralized osteoid surrounded by thick bundles of collagen fibers. The solid components of the mass multifocally displaced and infiltrated the hindlimb musculature, and femoral cortex and medullary cavity (Fig. 1E).

The cystic areas were lined by a single layer of cuboidal-to-columnal epithelial cells (resembling synoviocytes) that formed numerous plump villus-like proliferations protruding into the cyst lumen (Fig. 1F). In some areas, the lining epithelium was replaced by fibrin (Fig. 1F). Under the epithelium, a variable number of fibroblasts or fibrocytes, lymphocytes, macrophages with foamy cytoplasm, fibrin deposits, and a few heterophils were seen. In the lumen of the cysts, abundant homogeneous pale eosinophilic material (interpreted as synovial fluid) was seen, mixed with small numbers of degenerate epithelial cells, macrophages with foamy cytoplasm, heterophils, and fibrin. Based on the presence of solid and cystic components of the proliferation, the diagnosis of a synovial myxoma with cyst formation was made.

Histologic examination of other long bones revealed degenerative and regenerative changes. The cortical bone of the metaphysis and diaphysis had variable thinning and thickening, leading to an irregular bone outline (Fig. 2A) and reversal lines. Around the periosteum, multifocal-to-coalescing depositions of mature connective tissue, accumulations of chondrocytes, and proliferation of new-formed bone were visible (Fig. 2C). The islands of newly formed bone were multifocally covered by osteoblasts and/or by single osteoclasts in Howship lacunae. The number of trabeculae in the medullary cavity was generally diffusely reduced, and the remaining trabeculae were thin and often not mineralized. The surrounding skeletal musculature had degeneration and necrosis of myocytes and interstitial edema (Fig. 2D).

**Figure 2.**
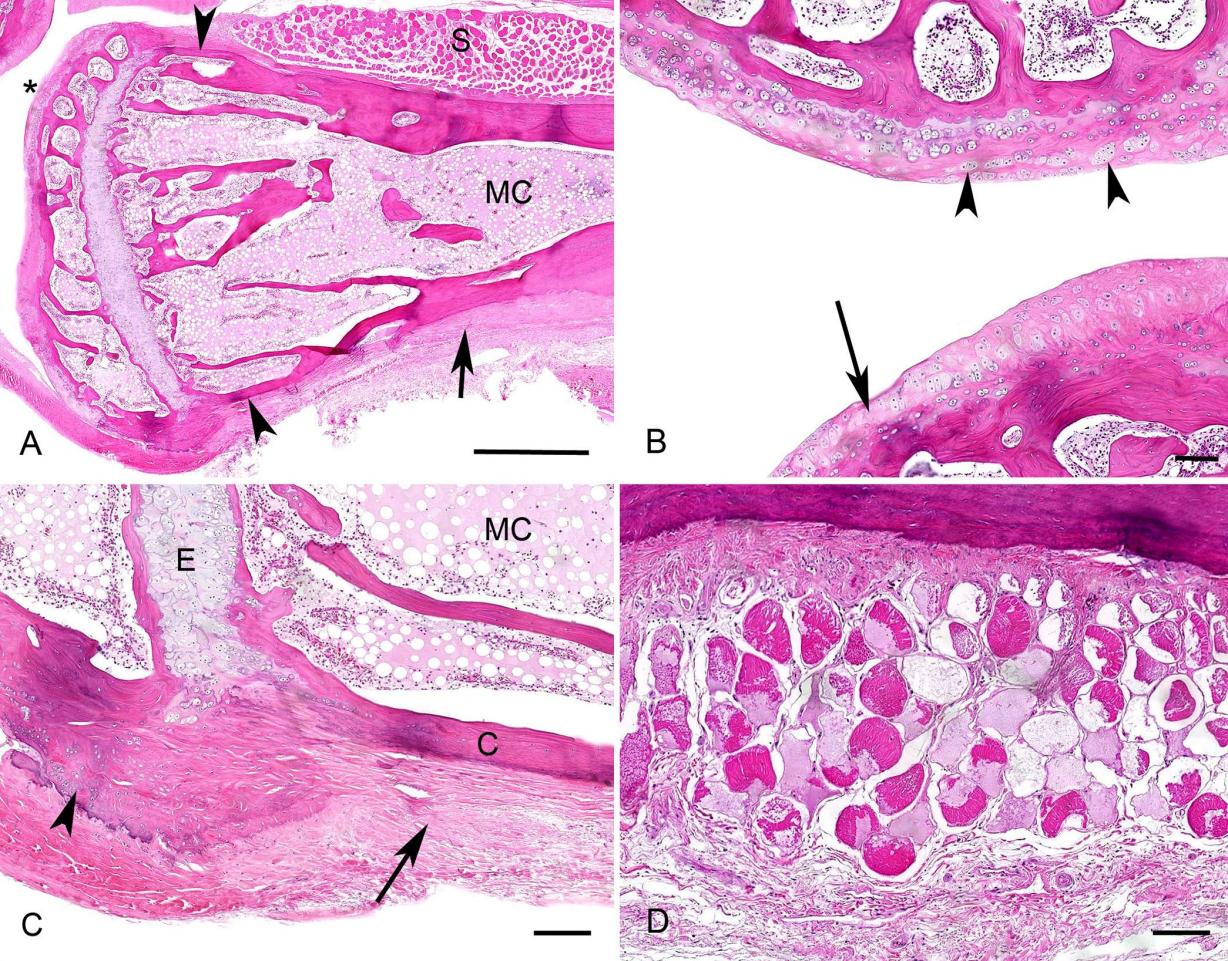
Metabolic bone and degenerative joint diseases in the right stifle joint and tibia in a central bearded dragon (*Pogona vitticeps*). **A.** Overview of the proximal end of the right tibia. The outline of the cortical bone is irregular, with severely thinned (arrowheads) and thickened areas. The medullary cavity (MC) has loss and thinning of bony trabeculae. Note the marked irregularity and thinning of the articular cartilage (asterisk) and the diffuse periosteal fibrosis (arrow). S = skeletal muscle. H&E. Bar = 500 µm. **B.** The articular cartilage of the tibia has severe thinning (arrow), and the remaining chondrocytes form small groups (arrowheads). H&E. Bar = 100 µm. **C.** Around the epiphysis (E), periosteal new bone formation is expanding the cortical bone (C); sclerosis (arrowhead) and fibrosis (arrow) are present. The medullary cavity (MC) is cell-poor and has diffuse serous atrophy of fat. H&E. Bar = 100 µm. **D.** The skeletal muscle around the long bones has severe degeneration and atrophy, with swelling, fragmentation, and loss of myofibers. H&E. Bar = 100 µm.

Articular surfaces also had diffuse thinning and fibrillation of the articular cartilage, eburnation of underlying bone, and periarticular fibrosis or multifocal new bone formation (Fig. 2B). Adipose tissue within the marrow cavity was cloudy and pale eosinophilic (gelatinous marrow transformation, or serous atrophy of fat), and numbers of hematopoietic stem cells were severely reduced (Fig. 2A, 2C). Additional histologic findings included a single hepatic cholangiocarcinoma and severe intestinal infestation with *Isospora* spp. and *Blastocystis* spp. In both kidneys, multifocal, moderate lymphoplasmacytic interstitial nephritis, accompanied by marked interstitial fibrosis, affected ~60% of the renal parenchyma. The aerobic and anaerobic bacterial culture of the left femoral joint revealed a low number of mixed-bacterial flora (*Staphylococcus epidermidis*, *S. warneri*, *Pseudomonas aeruginosa*, and gram-negative obligate anaerobes).

Myxomas and myxosarcomas are rare neoplasms of fibroblast origin, distinguished from other mesenchymal tumors by their abundant myxoid matrix rich in glycosaminoglycans. The literature does not provide defined histologic criteria for differentiating myxoma and myxosarcoma in domestic species. From a clinical point of view, the distinction between myxoma and myxosarcoma is academic, as they share a common biologic behavior. In the skin, increases in cellular density, nuclear pleomorphism, and mitoses generally favor the diagnosis of myxosarcoma, although this distinction is often subtle.^9^ In contrast, this terminology is not commonly applied when the neoplasm is located around joints, where, even in the case of local bone infiltration (with or without bone lysis), the term synovial myxoma is recommended.^3,14^ A retrospective study of submissions of captive lizard species concluded that, although historically considered rather rare in reptiles, neoplasia was diagnosed at a statistically significant higher proportion in bearded dragons and chameleons than in the other lizard species (e.g. leopard geckos, green iguanas).^11^ Myxoma or myxosarcoma in bearded dragons has predominantly been described in the skin and the periocular area (in one animal).^7,11,14^

In our case, the neoplasm had a striking gross and histologic appearance of cysts lined by cells resembling synoviocytes. Synovial myxomas in cats and dogs can occasionally have fluid-filled cysts lined by synovium; cyst formation might be part of the neoplastic process.^3^ However, as the cyst formation in our case was very prominent, a concurrent presence of a myxoma and synovial cysts, defined as benign periarticular swellings containing synovial fluid, cannot be excluded fully.^1^ In veterinary medicine, synovial cysts are most commonly described in the elbow joint of cats,^1,15^ in the vertebral column, and sporadically in the canine stifle and carpal joints.^4,5,16^ In humans, a proposed mechanism for developing synovial cysts is herniation of the synovial membrane through the joint capsule, possibly secondary to trauma or osteoarthritis.^17^ Interestingly, reports in humans and cats state a frequent co-occurrence of synovial cysts and myxomas.^1,2^ This finding may be related to neoplastic transformation of type B synoviocytes lining the cysts, indicating that these lesions might exist in a continuum rather than as 2 separate disease processes.^1,2^

In cats, synovial cysts of the elbow occur most frequently in elderly animals with degenerative joint disease (DJD).^2^ DJD has also been associated with increased joint laxity in cats, which is suspected to result in increased intraarticular pressure, leading to herniation of the synovium and, consequently, extraarticular cyst formation.^6,8,12^ The 3 consistent features of DJD, namely fibrillation, eburnation, and osteophyte formation, were also seen in our case. An association between DJD and articular myxomas is also described in humans.^1^

Bearded dragons are agamid lizards native to Australia that are well suited to captive conditions and have, therefore, become popular pets.^13^ They are diurnal and omnivorous, with the diet of adults based on plant matter.^13^ Lameness and diffuse swellings, especially along the long bones, are often related to MBD in this species, specifically nutritional secondary hyperparathyroidism (NSHP). The term MBD describes a collection of disorders in which the integrity and function of bones are affected; it is the most common cause of lameness and of skeletal and spinal abnormalities in reptile patients.^10^ MBD occurs commonly in diurnal herbivorous and insectivorous lizards and chelonians due to NSHP as a result of a deficiency in dietary calcium or vitamin D3, dietary imbalances of calcium:phosphorus ratios, or inadequate exposure to ultraviolet B radiation.^10^ Alternatively, chronic renal disease can lead to MBD due to renal secondary hyperparathyroidism (RSHP), characterized by hyperphosphatemia with low calcitriol levels.^10^

In our case, we observed histologic and radiographic evidence of MBD (reduction of cortical and trabecular bone, periosteal fibrosis) in all long bones examined. This animal had been acquired as an adult 3 y before presentation. Despite being offered mostly adequate husbandry recently, inadequate prior conditions could have influenced the health of this individual’s musculoskeletal system. This is also supported by the clinical history of the other co-housed individuals (chronic fractures, soft-tissue swellings). Additionally, the nephropathy could have also predisposed this individual to RSHP. Considering how frequently MBD occurs in reptiles and the rarity of the myxoma present in this individual, a causal relation of MBD and myxoma is unlikely.

The histologic picture of the lesions in the left femur is unusual as it is a neoplastic process combined with marked cyst formation that resulted in an overall highly variable appearance. Our findings support that myxomas with marked cyst formation can occur in the joints of reptiles and should be considered as clinical differentials for masses or swellings involving bones and joints. However, differential diagnoses, such as NSHP and fibrous osteodystrophy, trauma, gout, and infections, are more likely.
